# Testicular Development in Mice Lacking Receptors for Follicle Stimulating Hormone and Androgen

**DOI:** 10.1371/journal.pone.0035136

**Published:** 2012-04-13

**Authors:** Peter J. O’Shaughnessy, Ana Monteiro, Margaret Abel

**Affiliations:** 1 College of Medical, Veterinary and Life Sciences, Institute of Biodiversity, Animal Health and Comparative Medicine, University of Glasgow, Glasgow, United Kingdom; 2 Department of Human Anatomy and Genetics, University of Oxford, Oxford, United Kingdom; Clermont Université, France

## Abstract

Post-natal testicular development is dependent on gonadotrophin and androgen stimulation. Follicle stimulating hormone (FSH) acts through receptors (FSHR) on the Sertoli cell to stimulate spermatogenesis while androgens promote testis growth through receptors (AR) on the Sertoli cells, Leydig cells and peritubular myoid cells. In this study we have examined the effects on testis development of ablating FSHRs (FSHRKO mice) and/or ARs ubiquitously (ARKO mice) or specifically on the Sertoli cells (SCARKO mice). Cell numbers were measured using stereological methods. In ARKO mice Sertoli cell numbers were reduced at all ages from birth until adulthood. FSHR ablation also caused small reductions in Sertoli cell numbers up to day 20 with more marked effects seen in the adult. Germ cell numbers were unaffected by FSHR and/or AR ablation at birth. By day 20 ubiquitous AR or FSHR ablation caused a marked reduction in germ cell numbers with a synergistic effect of losing both receptors (germ cell numbers in FSHRKO.ARKO mice were 3% of control). Germ cell numbers in SCARKO mice were less affected. By adulthood, in contrast, clear synergistic control of germ cell numbers had become established between the actions of FSH and androgen through the Sertoli cells. Leydig cell numbers were normal on day 1 and day 5 in all groups. By day 20 and in adult animals total AR or FSHR ablation significantly reduced Leydig cell numbers but Sertoli cell specific AR ablation had no effect. Results show that, prior to puberty, development of most testicular parameters is more dependent on FSH action than androgen action mediated through the Sertoli cells although androgen action through other cells types is crucial. Post-pubertally, germ cell numbers and spermatogenesis are dependent on FSH and androgen action through the Sertoli cells.

## Introduction

Post-natal development of the testis depends on the action of the gonadotrophins follicle stimulating hormone (FSH) and luteinising hormone (LH) which are secreted by the pituitary gland. FSH acts directly on the Sertoli cells through the FSH-receptor (FSHR) while LH acts to stimulate androgen secretion by the Leydig cells. This androgen then acts on all cells expressing the androgen receptor (AR) in the testis; primarily the Sertoli cells, peritubular myoid cells and the Leydig cells [1]–[3]. The generation of transgenic mouse models which lack gonadotrophins, gonadotrophin receptors or androgen receptors has established the role that these factors play in the development of normal testicular function [2], [4]–[8]. Results from these studies have shown that FSH is required for normal Sertoli cell and germ cell numbers while androgen action through the Sertoli cells is essential for spermatocyte progression through meiosis and is reported to be required for normal development of Leydig cell numbers [9]. These hormones do not act in isolation, however, and generation of double knock-out animals lacking both the FSHR and AR on the Sertoli cells has shown that FSH and androgen act synergistically to stimulate completion of meiosis and entry into spermiogenesis in the adult animal [10]. While the role of FSH and androgen in the adult animal is now well documented the comparative importance of each hormone, and of hormone interactions, during development is less clear. This may be of particular relevance to germ cell development as the first wave of spermatogenesis appears to differ from subsequent waves [11]. Consequently, in this study we have examined testicular development in mice lacking the FSHR and/or ARs in the Sertoli cells. For comparison we have also examined the developmental effect of ubiquitous AR deletion, with or without loss of the FSHR.

## Results

Animals referred to in this study are ARKO mice (ubiquitous deletion of the AR), SCARKO mice (Sertoli-cell specific deletion of the AR), FSHRKO mice (ubiquitous deletion of the FSHR) and combinations thereof.

### Testis Morphology on Day 1

**Figure 1:**
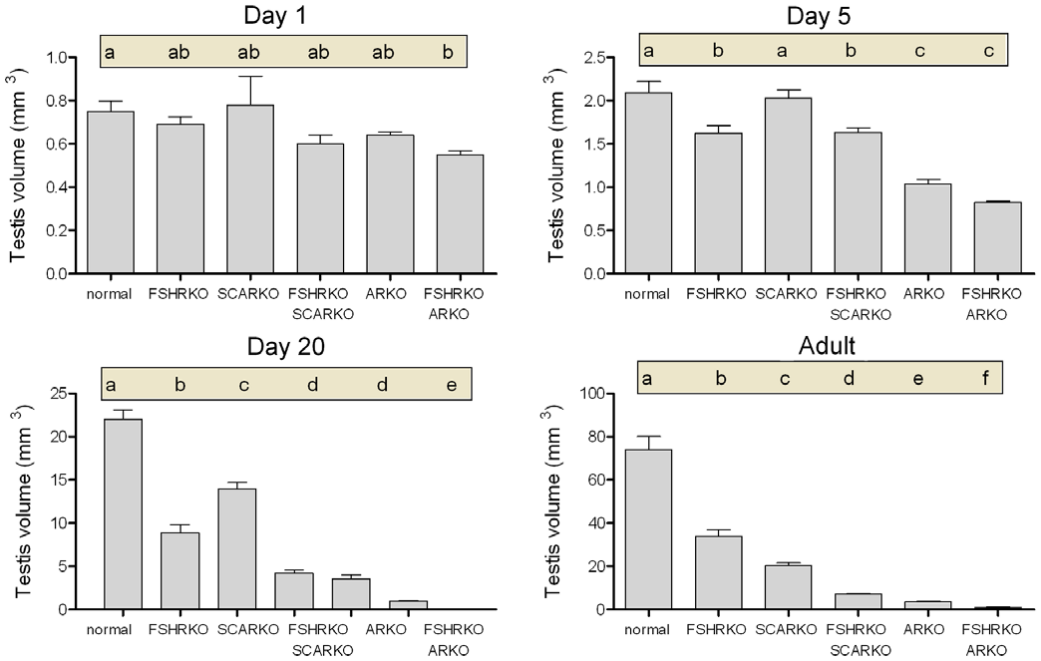
Role of FSHR and AR in determining testis volume during development. Testis volume was measured in normal, FSHRKO, SCARKO, FSHRKO.SCARKO, ARKO and FSHRKO.ARKO mice during post-natal development. Results show the mean ± sem of 3 to 6 animals per group at each individual age. Data was analysed initially by 2 factor anova and then by t-test using the pooled variance. Results of the t-test analysis are shown above the bars, groups which do not share a common letter are significantly different. The anova at each age showed: Day 1 - significant effect of FSHR knockout; Day 5 - significant effects of FSHR and AR/SCAR knockouts; Day 20 - significant effects of FSHR and AR/SCAR knockouts; Adult - significant interaction between factors.

**Figure 2 pone-0035136-g002:**
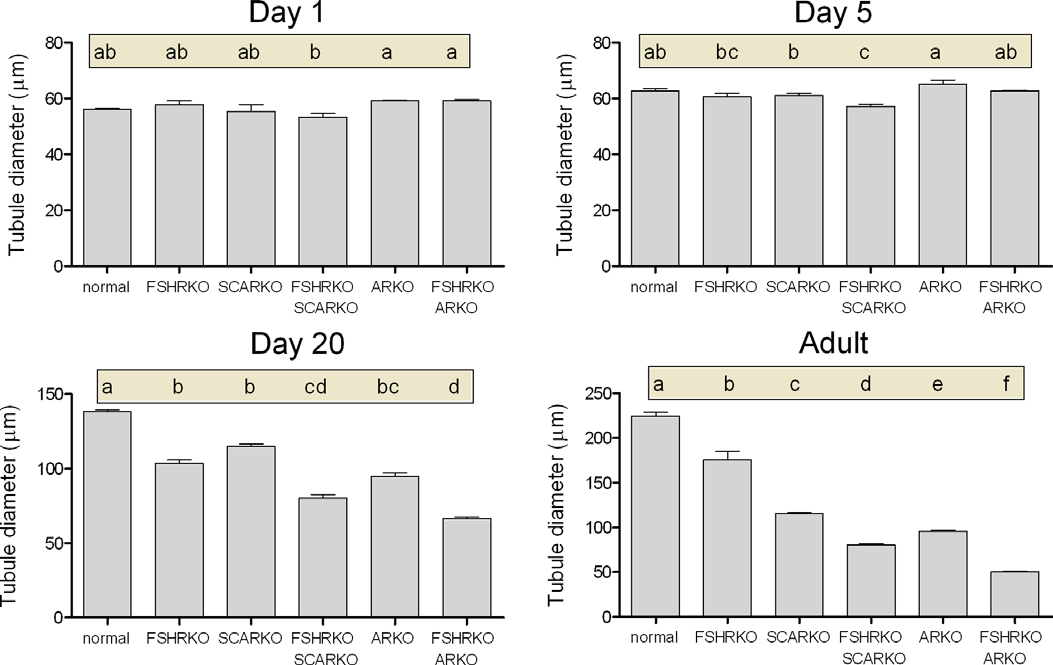
Role of FSHR and AR in determining tubule diameter during development. Tubule diameter was measured in normal, FSHRKO, SCARKO, FSHRKO.SCARKO, ARKO and FSHRKO.ARKO mice during post-natal development. Results show the mean ± sem of 3 to 6 animals per group at each individual age. Data was analysed initially by 2 factor anova and then by t-test using the pooled variance. Results of the t-test analysis are shown above the bars, groups which do not share a common letter are significantly different. The anova at each age showed: Day 1 - significant effect of AR/SCAR knockout; Day 5 - significant effects of FSHR and AR/SCAR knockouts; Day 20 - significant effects of FSHR and AR/SCAR knockouts; Adult - significant effects of FSHR and AR/SCAR knockouts.

**Figure 3 pone-0035136-g003:**
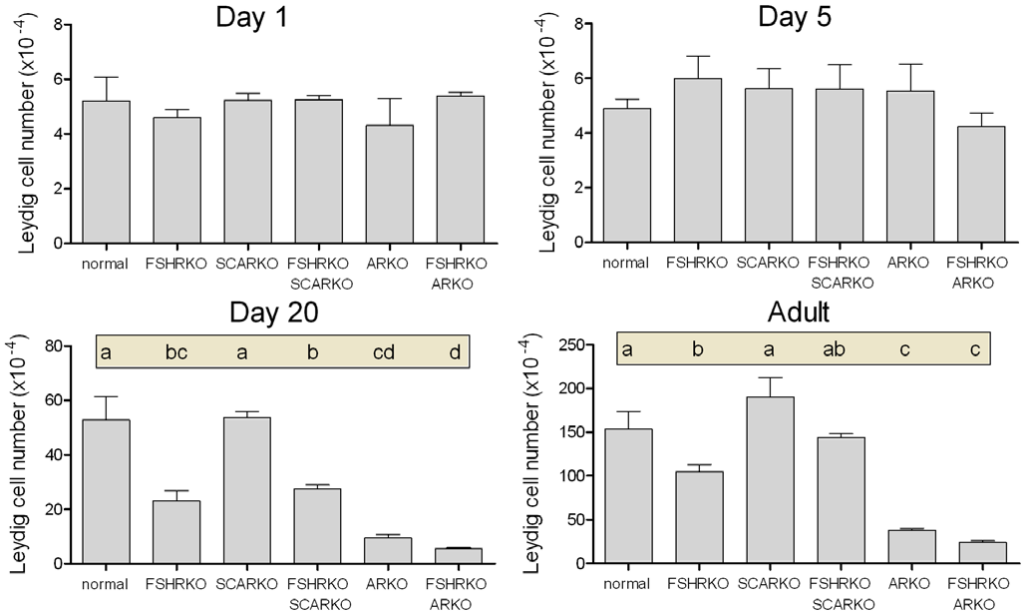
Role of FSHR and AR in determining Leydig cell number during development. Leydig cell number was measured in normal, FSHRKO, SCARKO, FSHRKO.SCARKO, ARKO and FSHRKO.ARKO mice during post-natal development. Results show the mean ± sem of 3 to 6 animals per group at each individual age. Data was analysed initially by 2 factor anova and then by t-test using the pooled variance. Results of the t-test analysis are shown above the bars, groups which do not share a common letter are significantly different. Where results of the anova were not significant no further analysis was carried out. The anova at each age showed: Day 1 - no significant differences; Day 5 - no significant differences; Day 20 - significant interaction between factors; Adult - significant effects of FSHR and AR/SCAR knockouts.

**Figure 4 pone-0035136-g004:**
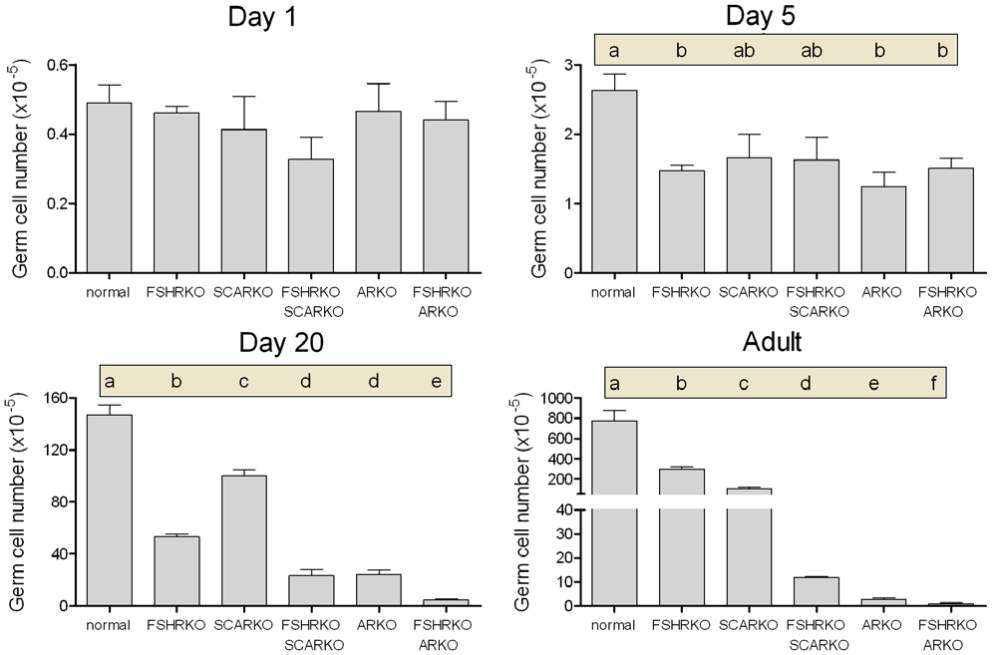
Role of FSHR and AR in determining germ cell number during development. Germ cell number was measured in normal, FSHRKO, SCARKO, FSHRKO.SCARKO, ARKO and FSHRKO.ARKO mice during post-natal development. Results show the mean ± sem of 3 to 6 animals per group at each individual age. Data was analysed initially by 2 factor anova and then by t-test using the pooled variance. Results of the t-test analysis are shown above the bars, groups which do not share a common letter are significantly different. Where results of the anova were not significant no further analysis was carried out. The anova at each age showed: Day 1 - no significant differences; Day 5 - significant interaction between factors; Day 20 - significant interaction between factors; Adult - significant interaction between factors.

**Figure 5 pone-0035136-g005:**
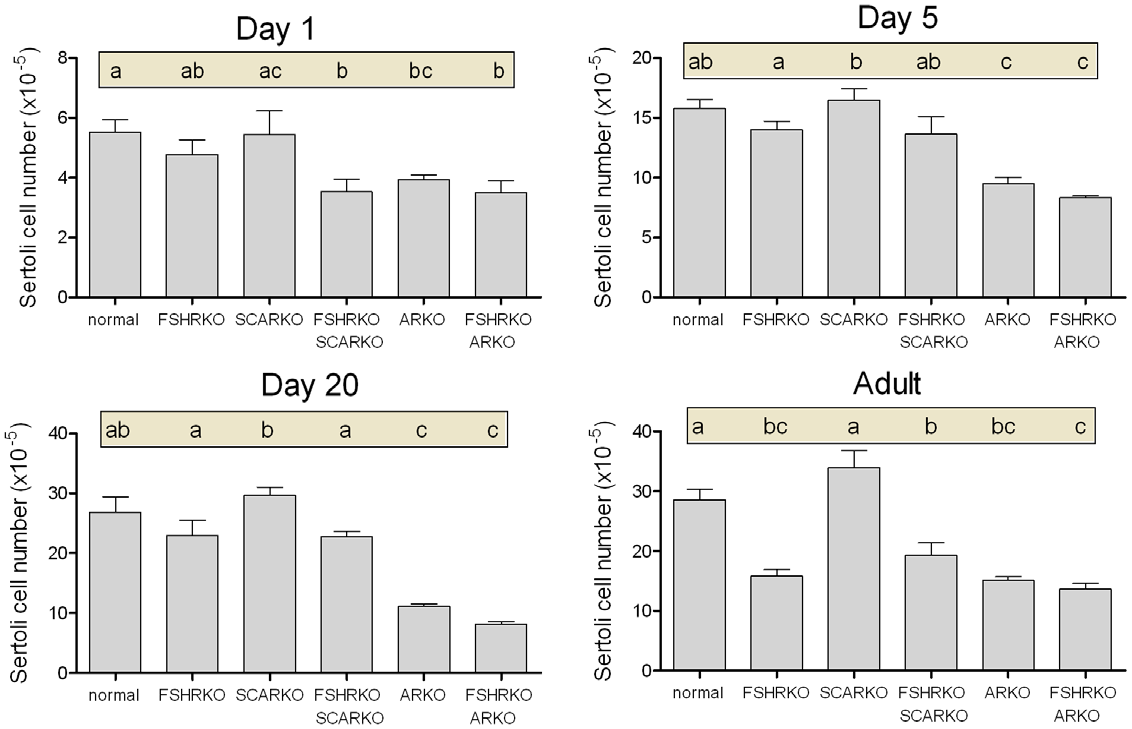
Role of FSHR and AR in determining Sertoli cell number during development. Sertoli cell number was measured in normal, FSHRKO, SCARKO, FSHRKO.SCARKO, ARKO and FSHRKO.ARKO mice during post-natal development. Results show the mean ± sem of 3 to 6 animals per group at each individual age. Data was analysed initially by 2 factor anova and then by t-test using the pooled variance. Results of the t-test analysis are shown above the bars, groups which do not share a common letter are significantly different. The anova at each age showed: Day 1 - significant effects of FSHR and AR/SCAR knockouts; Day 5 - significant effects of FSHR and AR/SCAR knockouts; Day 20 - significant effects of FSHR and AR/SCAR knockouts; Adult - significant interaction between factors.

**Figure 6:**
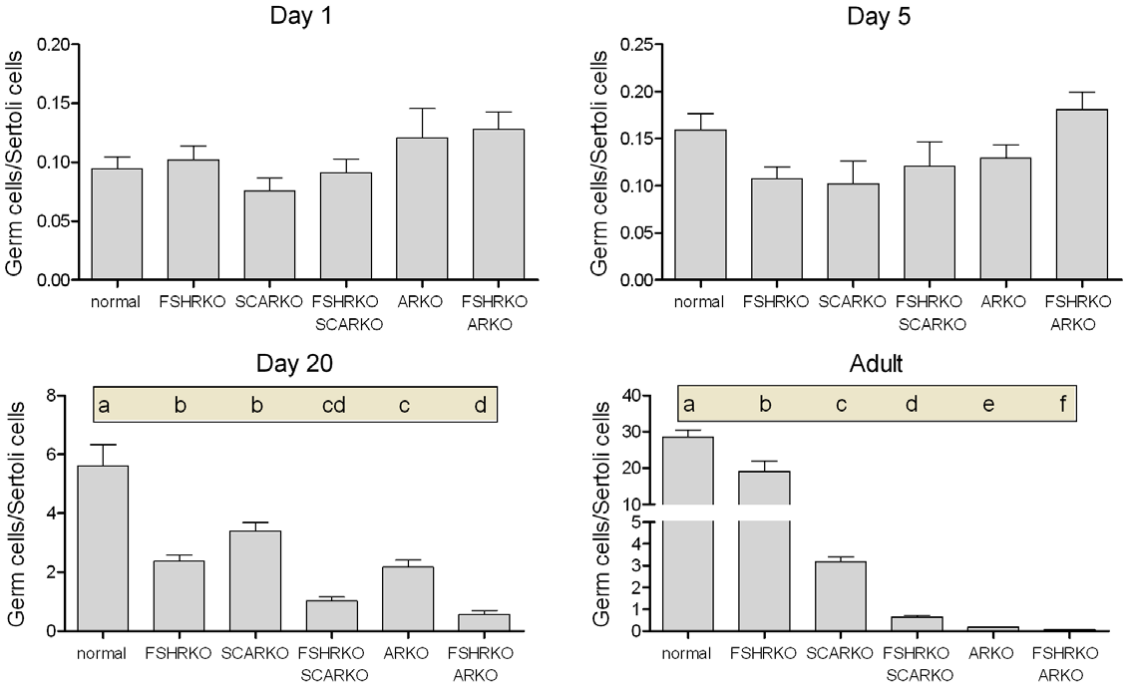
Role of FSHR and AR in determining germ cell to Sertoli cell ratio during development. The germ cell/Sertoli cell ration was determined in normal, FSHRKO, SCARKO, FSHRKO.SCARKO, ARKO and FSHRKO.ARKO mice during postnatal development. Results show the mean ± sem of 3 to 6 animals per group at each individual age. Data was analysed initially by 2 factor anova and then by t-test using the pooled variance. Results of the t-test analysis are shown above the bars, groups which do not share a common letter are significantly different. Where results of the anova were not significant no further analysis was carried out. The anova at each age showed: Day 1 - significant effects of AR/SCAR knockouts; Day 5 - no significant differences; Day 20 - significant effects of FSHR and AR/SCAR knockouts; Adult - significant interaction between factors.

On day 1 (day of birth) testes from mice lacking the FSHR (ie FSHRKO, FSHRKO.SCARKO and FSHRKO.ARKO mice) were significantly smaller than testes from animals with a functional FSHR (ie normal, SCARKO and ARKO mice) (Fig. 1 and statistical analysis in legend). Loss of the AR (either ubiquitously or in the Sertoli cells only) had no effect on testis size at this age. Morphologically the testes were very similar between groups although tubule diameter varied slightly (but significantly) depending on the presence or absence of a functional AR (Fig. 2). Leydig cell number and germ cell number did not vary significantly between groups (Figs. 3 and 4). Two factor analysis of variance (anova) showed that Sertoli cell number was significantly reduced overall by loss of the FSHR (Fig. 5) but there was no significant difference between normal and FSHRKO mice when compared directly. Sertoli cell number was reduced in ARKO testes at this age. Despite changes in Sertoli cell numbers there were no significant differences in the germ cell/Sertoli cell ratio at birth (Fig. 6).

### Testis Morphology on Day 5

**Figure 7:**
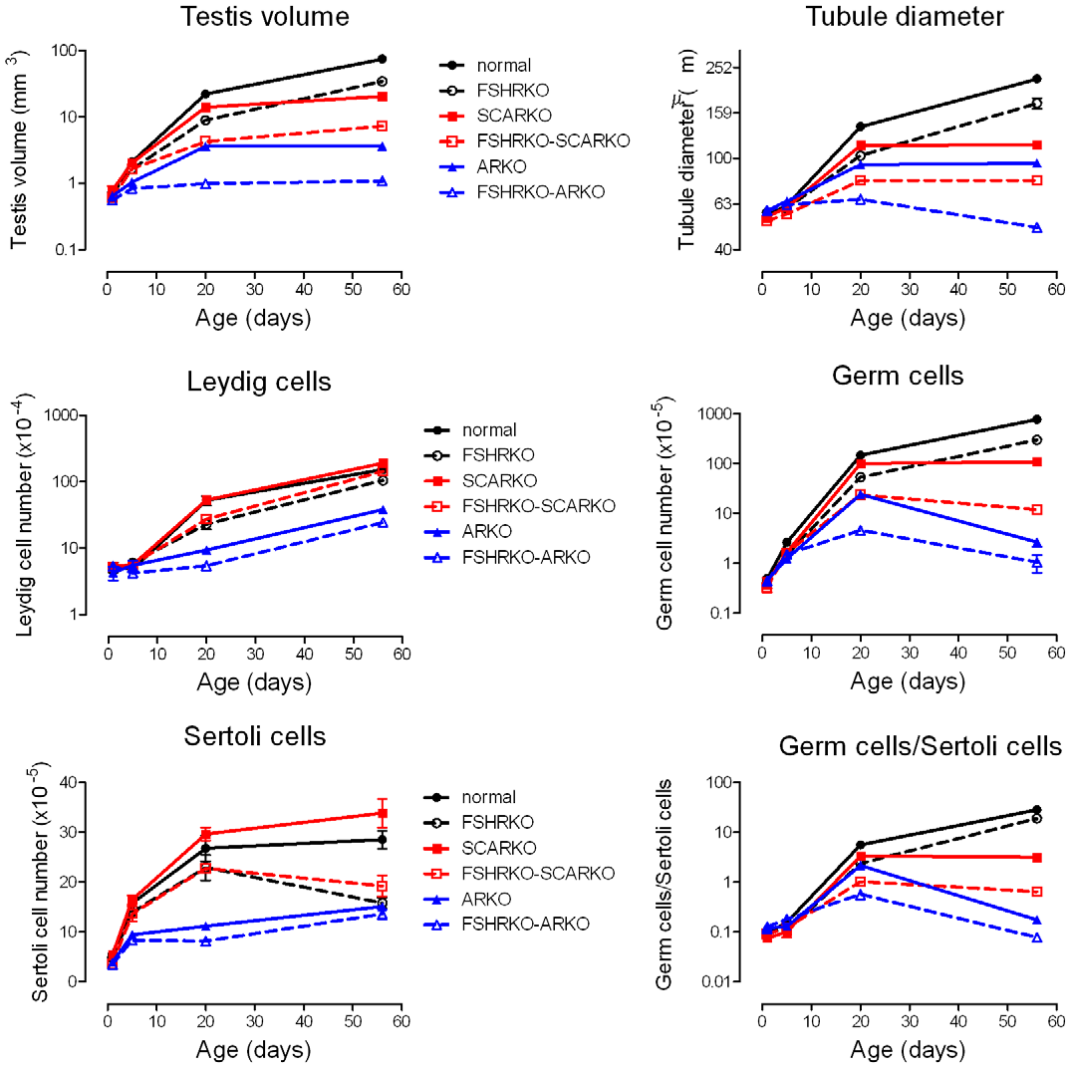
Summary data showing the role of FSHR and AR in testicular development. Data from figures 1 to [Fig pone-0035136-g002] [Fig pone-0035136-g003] [Fig pone-0035136-g004] [Fig pone-0035136-g005] 6 have been re-plotted to show developmental changes in testicular parameters and cell numbers in normal, FSHRKO, SCARKO, FSHRKO.SCARKO, ARKO and FSHRKO.ARKO mice. Note that for illustrative purposes the Y axis on all graphs, apart from Sertoli cells, is on a log scale. The mean ± sem is shown.

**Figure 8:**
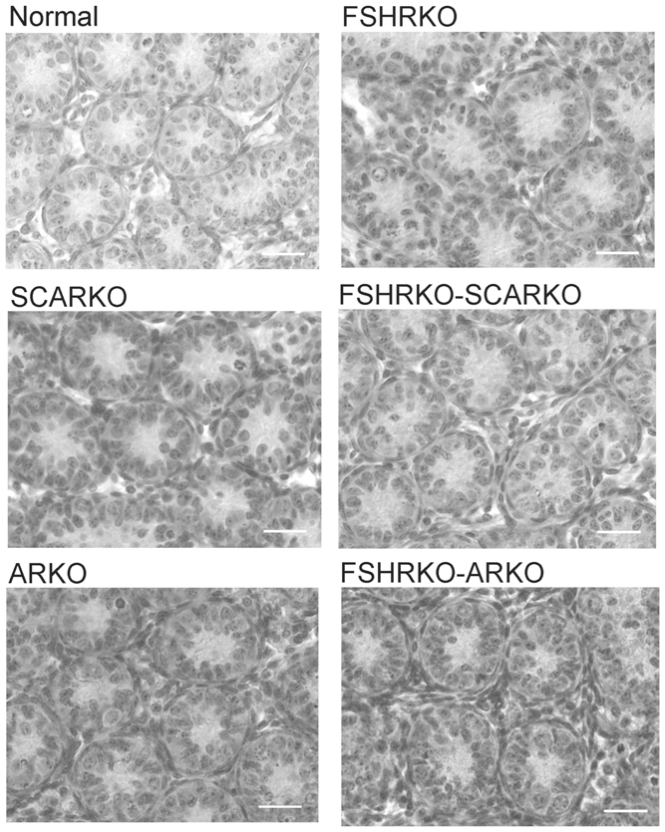
Testis morphology at day 5 in mice lacking the FSHR and/or AR. Testes from normal, FSHRKO, SCARKO, FSHRKO.SCARKO, ARKO and FSHRKO.ARKO mice at day 5 were fixed in Bouins and stained with H&E. The bar represents 30 µm.

By day 5 quite marked and significant differences in testis volumes were apparent with reduced volumes in all mice lacking the FSHR and in the ARKO groups (ARKO and FSHRKO.ARKO mice) (Fig. 1). There were also small but significant overall differences in tubule diameter with the smallest diameter apparent in FSHRKO.SCARKO mice (Fig. 2). Sertoli cell numbers were increased in all groups on day 5 compared to day 1 (Fig. 7) and, as on day 1, cell number was reduced by loss of the FSHR and in the ARKO group (Fig. 5). A reduction in germ cell number was also seen at this age in FSHRKO, ARKO and FSHRKO.ARKO mice (Fig. 4). The concomitant reduction in germ cells and Sertoli cells meant that there were no differences overall in the germ cell/Sertoli cell ratio (Fig. 6). Leydig cell number did not differ between groups at this age and were similar to Leydig cell numbers present at day 1 (Figs. 3 & 7). Testis morphology remained very similar between groups (Fig. 8) with germ cell migration to the basement membrane apparent in all groups.

### Testis Morphology on Day 20

**Figure 9:**
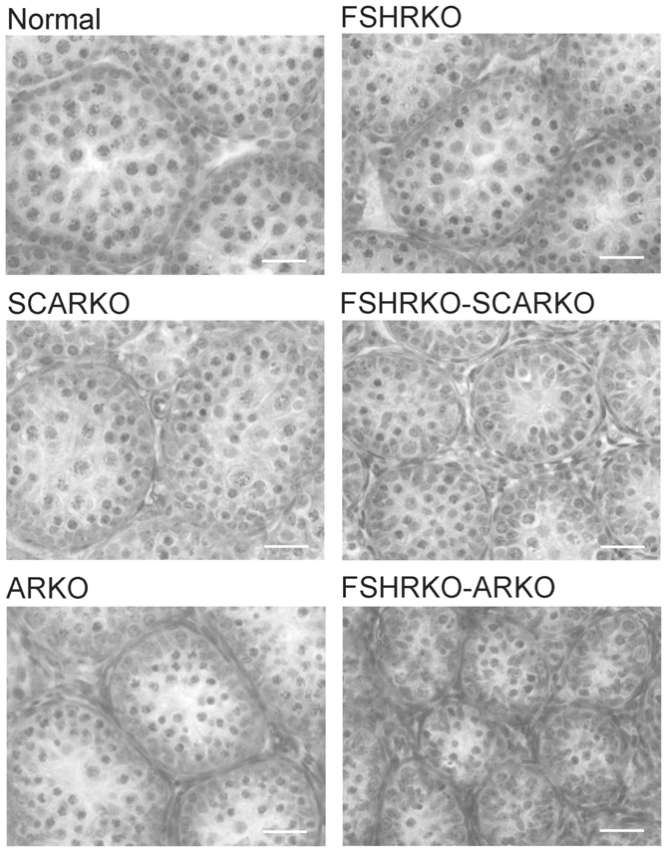
Testis morphology at day 20 in mice lacking the FSHR and/or AR. Testes from normal, FSHRKO, SCARKO, FSHRKO.SCARKO, ARKO and FSHRKO.ARKO mice at day 20 were fixed in Bouins and stained with H&E. The bar represents 30 µm.

On day 20 testis volume was significantly reduced in all experimental groups with the most marked effect seen in FSHRKO.ARKO mice (Fig. 1). Similar reductions in tubule diameter were also seen although these were less marked (Fig. 2). At this age testis morphology became distinctly different between most groups due, largely, to differences in development of spermatogenesis (Fig. 9). In normal, control mice the tubules contained large numbers of primary spermatocytes. The morphology of the FSHRKO and SCARKO mice was similar to control animals although spermatocyte numbers appeared reduced in the lumen of the SCARKO testis. In FSHRKO.SCARKO and ARKO mice spermatocytes were present in the tubules although their number was clearly reduced. In FSHRKO.ARKO mice germ cells were sparse although primary spermatocytes were present (Fig. 9). Stereological analysis confirmed that germ cell numbers were reduced in FSHRKO and SCARKO mice compared to control with the effect more marked in FSHRKO mice (Fig. 4). FSHRKO.SCARKO and ARKO mice had similar, low numbers of germ cells and in FSHRKO.ARKO mice germ cell numbers were reduced to 3% of control and 19% of ARKO mice. In all groups, germ cell number was significantly increased at day 20 compared to day 5 (Fig. 7). Sertoli cell numbers in normal, FSHRKO, SCARKO and FSHRKO.SCARKO mice increased from day 5 to day 20 but there was little change in cell numbers in ARKO and FSHRKO.ARKO mice (Figs. 5 & 7). Sertoli cell numbers remained significantly reduced in animals lacking the FSHR and in the ARKO group. The germ cell/Sertoli cell ratio was significantly reduced in all knockout groups at day 20 although the effects were most marked in the FSHRKO.SCARKO and FSHRKO.ARKO groups (Fig. 6) Compared to day 5 there was an increase in germ cell/Sertoli cell ratio in all groups at day 20 (Figs. 6 & 7). Leydig cell numbers increased markedly from day 5 to day 20 in normal, FSHRKO, SCARKO and FSHRKO.SCARKO mice but increased less than 2-fold in ARKO and FSHRKO.ARKO mice (Figs. 3 & 7). Overall, Leydig cell number at day 20 was reduced significantly in animals lacking the FSHR and in the ARKO group. There was no difference in Leydig cell number between normal and SCARKO mice.

### Testis Morphology in the Adult

**Figure 10:**
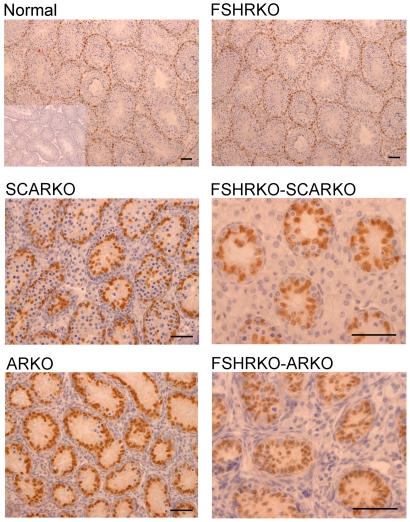
SOX9 expression in adult mice lacking the FSHR and/or AR. Testes from adult normal, FSHRKO, SCARKO, FSHRKO.SCARKO, ARKO and FSHRKO.ARKO mice were fixed in Bouins and immunohistochemistry was used to identify and label Sertoli cells using antibody against SOX9 (brown stain). The bar represents 50 µm.

**Figure 11:**
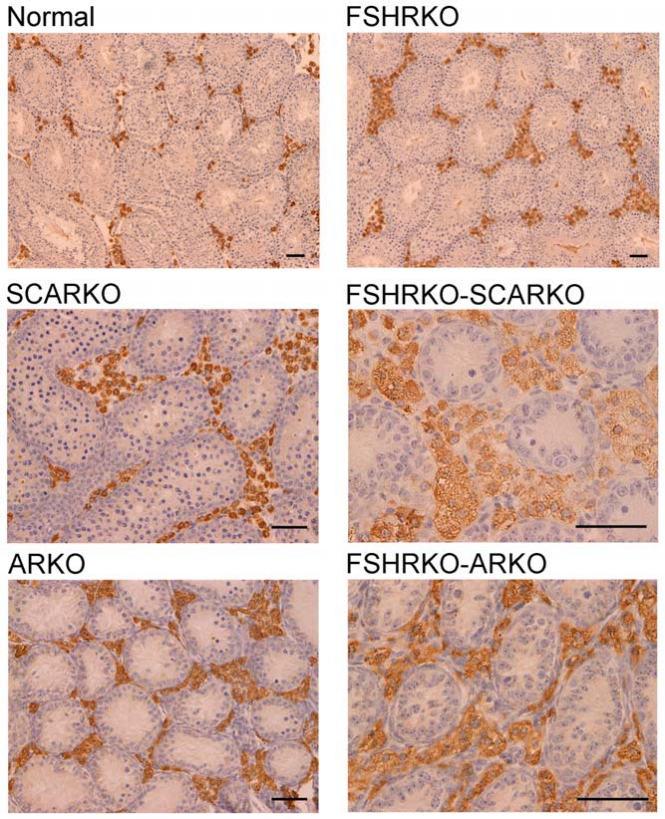
CYP11A1 expression in adult mice lacking the FSHR and/or AR. Testes from adult normal, FSHRKO, SCARKO, FSHRKO.SCARKO, ARKO and FSHRKO.ARKO mice were fixed in Bouins and immunohistochemistry was used to identify and label Leydig cells using antibody against CYP11A1 (brown stain). The bar represents 50 µm.

In adult mice testis volume was reduced by both loss of the FSHR and the AR and there was a synergistic interactive effect of losing both receptors so that the volume of the FSHRKO.ARKO mouse testis was reduced to 1.4% of control values (Fig. 1). Tubule diameter in both normal and FSHRKO mice increased markedly compared to day 20 but there was no change in either SCARKO or ARKO groups (Figs. 2 & 7). The increase in diameter in normal and FSHRKO mice was due to the development of complete spermatogenesis in these groups (Figs. 10 & 11) and the associated large increases in germ cell numbers in both groups (Figs. 4 & 7). Germ cell number in SCARKO mice did not change between 20 days and adulthood but decreased by 50% in FSHRKO.SCARKO mice and by 90% and 80% in ARKO and FSHRKO.ARKO mice respectively (Fig. 7). Sertoli cell number did not change markedly from 20 days to adulthood in most groups but decreased in FSHRKO mice (Figs. 5 & 7). Maintenance of Sertoli cell numbers but loss of germ cells meant that there was a marked decrease in the germ cell/Sertoli cell ratio in FSHRKO.SCARKO, ARKO and FSHRKO.ARKO mice (Fig. 6). This was reflected in the increased relative density of staining for the Sertoli cell marker SOX9 in the tubules of these mice (Fig. 10). Leydig cell numbers increased 3- to 5-fold in all groups between 20 days and adulthood so that the relationships between the groups did not change markedly compared to day 20 (Figs. 3 & 7). Leydig cell morphology did not differ markedly between groups and all showed abundant interstitial CYP11A1 staining (Fig. 11). The apparent hyperplasia of Leydig cells seen in Fig. 11 in, for example, the FSHRKO.SCARKO and the FSHRKO.ARKO mice is caused by the marked reduction in testis volume in these animals which compacts the Leydig cells into larger groups.

## Discussion

The role that FSH and androgen play in development and maintenance of testicular function has been the subject of continued study since the pioneering work of Smith, Greep and others in the 1920s and 1930s [12], [13]. Our current understanding is that, in the adult animal, FSH increases spermatogonial numbers and entry of these cells into meiosis while androgens increase the number of germ cell entering meiosis and, critically, enable these cells to complete meiosis [2], [10], [14], [15]. Adult FSHRKO mice are fertile [5], [6], albeit with reduced germ cell numbers, but SCARKO and ARKO mice are infertile [2] and it is clear, therefore, that androgens are more important for maintaining spermatogenesis and production of fertile sperm in the adult animal. Interestingly, results from this study show that the effects of FSHR ablation in the prepubertal animals were more marked than the effects of Sertoli cell AR ablation suggesting that FSH plays the more important role in regulating Sertoli cell function in pre-pubertal and early pubertal animals.

In mammals, the first wave of spermatogenesis to occur after birth develops in an environment which differs from subsequent waves in both the structure/activity of the testis and in the levels of circulating hormones. In addition, during the first wave many germ cells undergo apoptosis at the spermatocyte stage in an event which appears to be essential for subsequent normal adult spermatogenesis [16]. FSH normally acts to limit overall germ cell apoptosis during the first spermatogenic wave [17], [18] and if levels of the hormone are reduced by GnRH antagonists or by passive immunisation there is a marked increase in germ cell death. Significant loss of germ cells at 20d in the FSHRKO mouse is consistent, therefore, with this role of FSH during the first spermatogenic wave. Whether the increased apoptosis in the first wave of spermatogenesis in the FSHRKO mouse has an ongoing effect on germ cell numbers in the adult FSHRKO mouse is unclear. Androgens are also reported to reduce spermatocyte apoptosis in pre-pubertal rats [19] and this is likely to explain the decline in germ cell numbers at 20d in the SCARKO mouse. At 20 days, germ cell loss was less marked in the SCARKO mouse than in the FSHRKO mouse but the effects were additive with a significantly greater effect in the FSHRKO.SCARKO mouse. Germ cell loss in the ARKO mouse at 20 days was also particularly marked which is likely to be due to combined loss of AR in the Sertoli cells and the PTMCs [20]. This loss of germ cells in the ARKO mouse was also clearly exacerbated by deletion of the FSHR (FSHRKO.ARKO mice had 3% of control germ cell numbers at day 20) indicating that FSH and androgen action through the Sertoli cells and androgen action through the PTMC are all essential for pre-pubertal germ cell proliferation and survival.

In the adult, germ cell loss was significantly greater in SCARKO mice than in FSHRKO mice. This has been described previously [10] and the change from day 20 can be explained by the critical need for androgen action through the Sertoli cells in order to complete meiosis [2]. Germ cells do not normally complete meiosis until after 20 days and so the effect of Sertoli cell AR deletion does not become marked until this time. Interestingly, while germ cell numbers in the SCARKO mouse were maintained from 20d to adulthood and were increased in FSHRKO mice over the same period there was a 50% drop in germ cell numbers in FSHRKO.SCARKO mice. This would suggest that spermatogonia and/or spermatocytes become more dependent on hormone-stimulated Sertoli cell support after the first spermatogenic cycle.

Previous studies have shown that androgen action is essential for development of normal Sertoli cell numbers during late fetal and early postnatal development. The role of FSH during this period has been less clear however. Early studies reported that late fetal Sertoli cell proliferation in the rat is regulated by FSH [21] but initial studies of FSHRKO and FSHβKO mice found no difference in Sertoli cell number at birth compared to control mice [22]. Analysis of the current data does show a significant effect of FSHR ablation on Sertoli cell number in the neonate but this effect is small and can only be shown to be significant when exacerbated by loss of the AR. Sertoli cell numbers up to day 20 appear to be regulated largely by androgens and hormone-independent mechanisms with a small contribution coming from FSH. After day 20 there was a more marked loss of Sertoli cells in FSHRKO mice (an effect reported previously [22]) which suggests that FSH acts post-pubertally to prevent loss of Sertoli cells either through apopotosis [23] or detachment from the basement membrane.

During development two sequential populations of Leydig cells arise with the first, “fetal” population present until about post-natal day 7 when it is superseded by an “adult” population of cells [24], [25]. As shown previously, fetal Leydig cell numbers were not affected by loss of FSHR or AR while the adult Leydig cell population was significantly reduced in both FSHRKO and ARKO mice [9], [26], [27]. This is consistent with a role for both androgen and FSH in the development of adult Leydig cell number [28]. More surprisingly, we failed to see any difference in Leydig cell numbers between normal and SCARKO mice at 20 days or as adults. This is in contrast to a previous study [9] and the reason for this apparent discrepancy is not entirely clear but may be due to the stereological methods used and, in particular, identification of the cells. In this study Leydig cells were identified by their morphology whereas De Gendt *et al* [9] used an immunohistochemical stain for HSD3B. Any discrepancy between morphology and HSD3B expression would lead to a discrepancy in cell numbers. Alternatively, the SCARKO mice in this study are on a different background to those used previously [9] which may have affected the Leydig cell response to loss of Sertoli cell ARs. Our data would suggest that development of a full-sized population of Leydig cells is dependent on androgens but that those androgens do not act primarily through the Sertoli cells. In mice lacking ARs in the peritubular myoid cells the total Leydig cell number is reported to be normal [20] suggesting that androgens probably act directly on the Leydig cells to regulate cell number.

It is well established that FSH and androgen are required throughout post-natal life for normal testicular structural development and results from the ARKO mouse show that androgens overall are more important at all stages of post-natal development. With respect to actions mediated through the Sertoli cells, however, results reported here show that FSH plays a greater role in establishing most parameters of testicular development prior to puberty. The most marked effect was on the germ cells and changes in other measures of testicular development such as volume, tubule diameter and germ/Sertoli cell ratio probably stem from altered germ cell numbers. After puberty androgens become more important for germ cell development although androgen effects on somatic cell development do not appear to be mediated primarily through the Sertoli cells. Comparison of FSHRKO, SCARKO and ARKO mice with the double knockouts shows that for most of the measured parameters the actions of FSH and androgen are additive or synergistic. This suggests that the two hormones are acting at different parts of the developmental pathway for each parameter and is consistent with what we know about FSH and androgen action.

## Materials and Methods

### Ethics Statement

All mice were bred and all procedures were carried out under UK Home Office Licence and with the approval of the University of Oxford Committee on Animal Care and Ethical Review.

### Animals and Treatments

ARKO, SCARKO, FSHRKO and FSHRKO.SCARKO mice were generated as previously described [10]. FSHRKO.ARKO mice were produced by interbreeding animals carrying the PGK1-Cre transgene with animals carrying the AR^fl^ allele and with FSHRKO mice. The control animals for these studies were are described previously [10]. Animals were killed on day 1 (day of birth), day 5, day 20 or as adults (8 weeks) and testes fixed overnight in Bouin’s solution. Animals were genotyped by PCR as previously described [2], [29].

### Stereology

For stereological analysis, testes were embedded in Technovit 7100 resin, cut into sections (20μm), and stained with Harris’ hematoxylin. The total testis volume was estimated using the Cavalieri principle [30]. The optical disector technique [31] was used to count the number of Sertoli cells, Leydig cells and germ cells in each testis. Each cell type was identified by previously described criteria [32]–[34]. Briefly, Sertoli cells were recognised in neonates by position and by their irregular-shaped nucleus and lack of apparent cytoplam and in older animals by their nuclear shape and tripartite nucleolus. Leydig cells were recognised by their position, round nucleus and relatively abundant cytoplasm. Representative cells are shown in Figure S1. The numerical density of each cell type was estimated using an Olympus BX50 microscope fitted with a motorized stage (Prior Scientific Instruments, Cambridge, UK) and Stereologer software (Systems Planning Analysis, Alexandria, VA, USA). Stereological data for adult normal, FSHRKO, SCARKO and FSHRKO.SCARKO animals in this study includes data from a previous study [10] plus additional animals.

### Immunohistochemistry

For immunohistochemistry, testes were embedded in paraffin and sections (5 µm) were mounted on glass slides, dewaxed, and rehydrated. Sections were incubated with anti-CYP11A1 (rabbit polyclonal, donated by Dr AH Payne, Stanford) or anti-SOX9 (rabbit polyclonal, AB5535, Millipore, Watford, UK) after heat-induced antigen retrieval. Bound primary antibody was detected using a peroxidase-conjugated secondary antibody, followed by a fluorescyl-tyramide amplification step with visualization using 3,3-diaminobenzidine tetrahydrochloride (Dako UK Ltd., Cambridgeshire, UK). For negative control samples, non-immune serum replaced primary antiserum.

### Statistical Analysis

Data were analysed using two factor anova on a 3x2 design with normal vs SCARKO vs ARKO as one factor and the absence of functional FSHR (ie FSHRKO) as the other factor. A significant FSHRKO effect indicates that there is an overall effect (across normal, SCARKO and ARKO groups) of loss of the FSHR. A significant SCAR/AR effect indicates a difference between normal, SCARKO and ARKO groups while a significant interaction between the factors means that the effect of double knockouts is not simply an additive effect of each gene knockout alone. To show whether differences between individual groups were significant t-tests were employed using the pooled variance from the anova. Data were log transformed where appropriate to avoid heterogeneity of variance.

## Supporting Information

Figure S1 **Identification of cells for stereological analysis.** The image shows a section from a day 20 normal mouse testis. The white arrows indicate Sertoli cells while the black arrows indicate Leydig cells. The cells were recognised by their position (in a tubule or in the interstitium) and by their nuclear shape and visible cytoplasmic abundance.
(TIF)
