# Supplementary figures and images for: Testicular Development in Mice Lacking Receptors for Follicle Stimulating Hormone and Androgen
Source: PLoS One. 2012 Apr 13;7(4):e35136. doi: 10.1371/journal.pone.0035136 (PMC3325994; doi:10.1371/journal.pone.0035136)

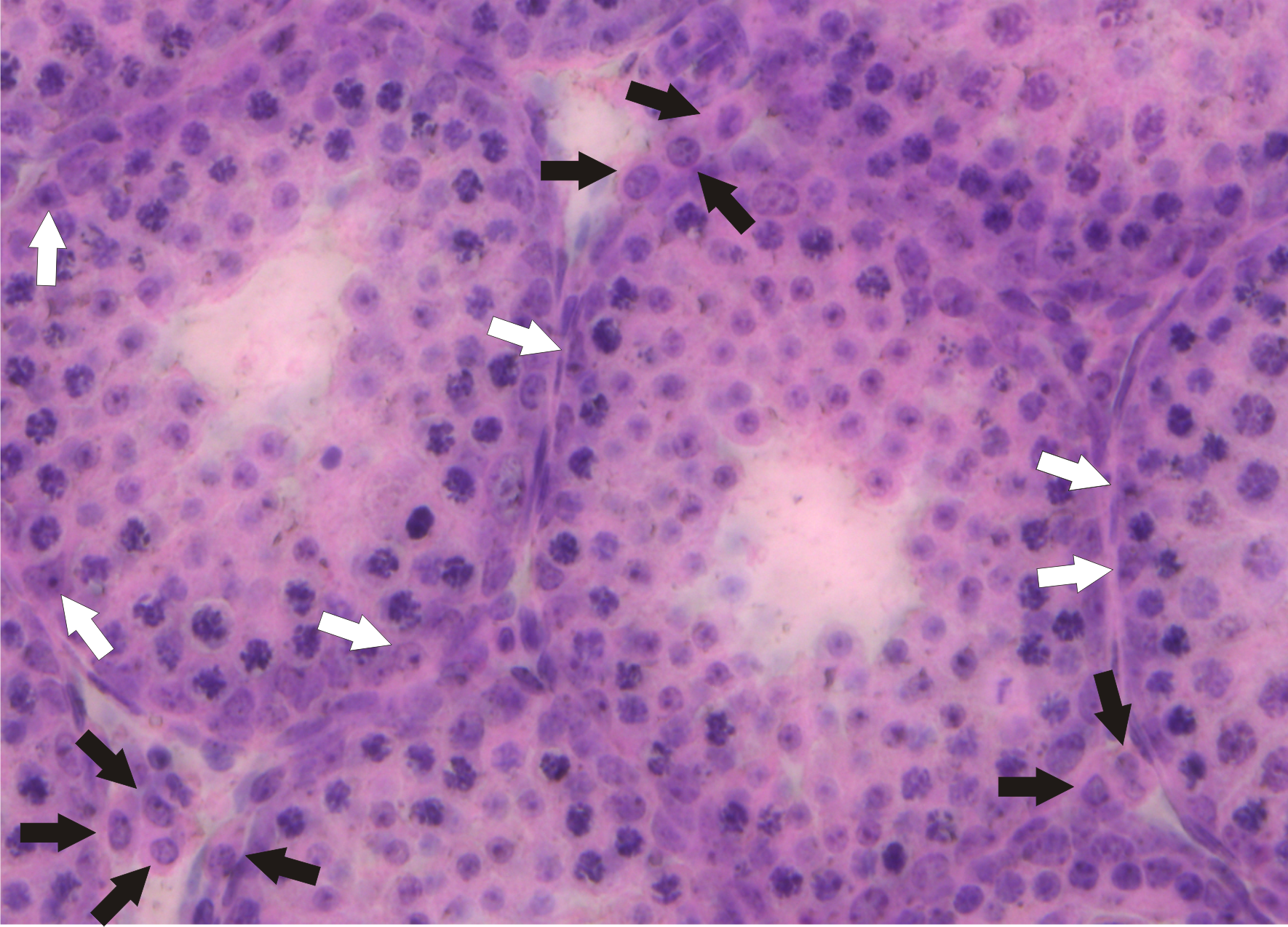

Supplement: Figure S1 — Identification of cells for stereological analysis. The image shows a section from a day 20 normal mouse testis. The white arrows indicate Sertoli cells while the black arrows indicate Leydig cells. The cells were recognised by their position (in a tubule or in the interstitium) and by their nuclear shape and visible cytoplasmic abundance. (TIF) [file pone.0035136.s001.tif]
